# NAD(P)H Oxidase Activity in the Small Intestine Is Predominantly Found in Enterocytes, Not Professional Phagocytes

**DOI:** 10.3390/ijms19051365

**Published:** 2018-05-04

**Authors:** Randall L. Lindquist, Jannike Bayat-Sarmadi, Ruth Leben, Raluca Niesner, Anja E. Hauser

**Affiliations:** 1 Deutsches Rheumaforschungszentrum Berlin, AG Immunodynamics, 10117 Berlin, Germany; Randall.Lindquist@drfz.de (R.L.L.); sarmadi@drfz.de (J.B.-S.); 2 Deutsches Rheumaforschungszentrum Berlin, AG Biophysical Analytics, 10117 Berlin, Germany; ruth.leben@drfz.de (R.L.); niesner@drfz.de (R.N.); 3 Department of Rheumatology and Clinical Immunology, Charité—Universitätsmedizin, 10117 Berlin, Germany

**Keywords:** mucosal immunology, fluorescence lifetime imaging, intravital imaging, metabolism, NADPH oxidase

## Abstract

The balance between various cellular subsets of the innate and adaptive immune system and microbiota in the gastrointestinal tract is carefully regulated to maintain tolerance to the normal flora and dietary antigens, while protecting against pathogens. The intestinal epithelial cells and the network of dendritic cells and macrophages in the lamina propria are crucial lines of defense that regulate this balance. The complex relationship between the myeloid compartment (dendritic cells and macrophages) and lymphocyte compartment (T cells and innate lymphoid cells), as well as the impact of the epithelial cell layer have been studied in depth in recent years, revealing that the regulatory and effector functions of both innate and adaptive immune compartments exhibit more plasticity than had been previously appreciated. However, little is known about the metabolic activity of these cellular compartments, which is the basic function underlying all other additional tasks the cells perform. Here we perform intravital NAD(P)H fluorescence lifetime imaging in the small intestine of fluorescent reporter mice to monitor the NAD(P)H-dependent metabolism of epithelial and myeloid cells. The majority of myeloid cells which comprise the surveilling network in the lamina propria have a low metabolic activity and remain resting even upon stimulation. Only a few myeloid cells, typically localized at the tip of the villi, are metabolically active and are able to activate NADPH oxidases upon stimulation, leading to an oxidative burst. In contrast, the epithelial cells are metabolically highly active and, although not considered professional phagocytes, are also able to activate NADPH oxidases, leading to massive production of reactive oxygen species. Whereas the oxidative burst in myeloid cells is mainly catalyzed by the NOX2 isotype, in epithelial cells other isotypes of the NADPH oxidases family are involved, especially NOX4. They are constitutively expressed by the epithelial cells, but activated only on demand to ensure rapid defense against pathogens. This minimizes the potential for inadvertent damage from resting NOX activation, while maintaining the capacity to respond quickly if needed.

## 1. Introduction

Although located inside the body, the GI gastro-intestinal (GI) tract is considered the largest body surface. Its physiological function, the digestion and absorption of water and nutrients, leads to a continuous exposure to foreign antigens. The GI tract hosts a large number of commensal microorganisms, most of which are mutualistic symbionts. However, the GI tract is also a preferential entry site of pathogens, as the luminal surface and host tissue are only separated by one layer of epithelial cells, the enterocytes [1]. Thus, the gut epithelium must provide a barrier to the environment, while nutrients are being taken up actively. At the same time, tolerance to dietary antigens and commensal microorganisms has to be maintained, as hypersensitivity to dietary- and commensal-derived antigens can lead to inflammatory disorders like Celiac disease or Inflammatory Bowel Disease (IBD) [1]. To fulfill this task, the GI tract harbors the body’s largest fraction of immune cells. The mechanisms that provide tolerance and protection involve the fine-tuning between regulatory and effector functions of the cell types regulating gut immunity.

Besides constituting the physical barrier between luminal content and the host tissue, intestinal epithelial cells also have crucial functions in immune regulation and actively defend the organism against microbial invasion. For example, goblet cells, which represent specialized epithelial cells, produce a thick layer of mucus that is impenetrable for most microorganisms. Antimicrobial proteins like regenerating islet-derived protein 3 gamma (REGIIIγ), defensins, cathelicidins and lysozyme, which further reinforce the barrier function, are produced by epithelial cells in intestinal villi and by Paneth cells in the crypt region [2]. To sense microbes, epithelial cells express a set of pattern recognition receptors, and they respond to microbial encounters by expressing cytokines and signaling molecules [2]. For example, epithelial cells produce B-cell activating factor (BAFF) and a proliferation-inducing ligand (APRIL) [3], which can induce T cell-independent class-switching of B cells to IgA in response to microbial stimulation [4,5]. Commensal- dependent expression of thymic stromal lymphopoietin (TSLP), transforming-growth factor β (TGF-β) and the vitamin A metabolite retinoic acid by epithelial cells promote the development of tolerogenic dendritic cells [6,7,8]. Interleukin (IL)-15 and IL-7, two cytokines important for the maintenance of intraepithelial lymphocytes (IEL) [9,10,11], are induced in epithelial cells in response to bacterial exposure. Epithelial cells express E-cadherin, the ligand for αEβ7-integrin (CD103), which is present on IELs and crucial for retaining these lymphocytes in the intraepithelial compartment [12]. Furthermore, CD8αα^+^ IELs need the non-classical MHCI-like ligand thymus leukemia antigen (TLA) expressed by intestinal epithelial cells [13], in order to become activated.

One of the largest populations of macrophages in the body can be found in the intestinal lamina propria (LP) of the GI tract, where macrophages are located in tissue underneath the epithelium and throughout the villi [14]. Here they interact with epithelial cells and have been shown to produce mediators that promote renewal of the epithelium [15]. This strategic positioning allows these phagocytic cells to take up microbes that have passed through the epithelial barrier and thereby help to prevent infection. It has been shown that intestinal macrophages can extend protrusions through the epithelium to capture bacteria directly from the epithelial surface [16]. Intestinal macrophages have several unusual phenotypic features. Besides the classical macrophage markers F4/80, CD68 and CD11b, they also express MHCII and intermediate to high levels of CD11c and high levels of the fractalkine receptor CX3CR1 [17,18,19]. To detect and analyze intestinal macrophages in the present study, a reporter mouse for CX3CR1-expressing cells was used, which was generated by targeted insertion of GFP into the *CX3CR1* gene [20]. It has been shown that CX3CR1^+^ macrophages in the colon arise from bone marrow (BM) derived Ly6C^hi^ blood monocytes [17,21]. They express the chemokine receptor CCR2, which is important for the egress of these cells from the BM and enter the intestine via the blood [22]. In the LP, they further develop into Ly6C^lo^ monocytes, which downregulate CCR2 and upregulate CX3CR1. The development into mature, tissue resident macrophages involves increased expression of MHCII and CX3CR1, as well as downregulation of Ly6C [17,21]. Under steady-state conditions, CX3CR1^+^ macrophages in the lamina propria lack proliferative activity and are slowly but constantly replaced by incoming precursor cells that locally differentiate into mature macrophages. Under inflammatory conditions, Ly6C^hi^ monocytes, which further develop into CX3CR1^int^ monocytes, accumulate in the LP. Because they express inflammatory mediators such as TNFα, IL-6, IL-12, and IL-23 and are responsive to TLR-stimulation, these monocytes have been termed inflammatory monocytes, in contrast to CX3CR1^+^ macrophages which retain their anti-inflammatory characteristics even under inflammatory conditions [17,23,24].

Here, we analyze the activation of NADPH oxidases (the NOX enzyme family) in the intestine in vivo [25,26,27,28], by intravital microscopy. By measuring the endogenous fluorescence lifetime of NAD(P)H [29], and thereby exploiting the marked prolongation of NAD(P)H’s fluorescence lifetime upon binding of NAD(P)H to NOX as compared to other metabolic enzymes, we show that NOX enzymes are activated not only in CX3CR1^+^ phagocytes, but also in the gut epithelium in the steady state. Upon activation by chemical stimuli or bacterial products, NOX activity increases in both gut epithelial cells as well as in intestinal phagocytes.

## 2. Results

### 2.1. Dynamic and Functional Intravital Imaging in Villi of the Small Intestine

**Figure 1:**
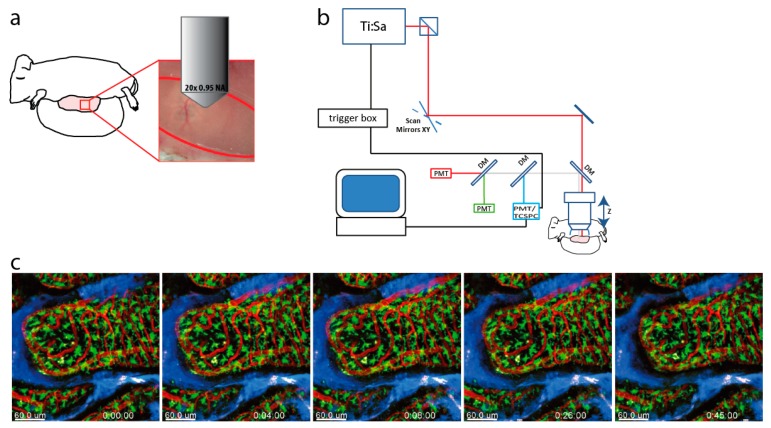
Intravital multi-photon fluorescence microscopy of the small intestine (**a**) Small intestine preparation for imaging: the small intestine of a mouse was exteriorized, its wall was carefully cut to avoid bleeding, placed on a heated stage for imaging and fixed by a thin layer of low-melting agarose. In this way intestinal villi were exposed and made accessible to the microscope objective lens. (**b**) Experimental setup: the beam of a 80 MHz pulsed titanium sapphire laser (Ti:Sa) at 760 nm is scanned by a galvoscanner over the sample (S). The laser beam is focused by a 20× water immersion NA 1.05 objective. Excitation and emission light are separated by a dichroic mirror (cut off 695 nm), the emission light is split by dichroic mirrors and interference filters and detected by photomultiplier tubes (PMT). The blue fluorescence (IF 466/40) is detected either by a time-correlated single photon counter (TCSPC) in a time-resolved manner or by a photomultiplier tube (PMT). (**c**) Time-lapse 3D fluorescence images of an intestinal villus: the resident macrophages in the lamina propria of a CX3CR1-GFP mouse are displayed in green, the blood flow labeled by rhodamine dextran is shown in red and the epithelium labeled by 7-Amino-4-Chlormethylcoumarin (CMAC) is shown in blue. The time lapse series and Supplementary Movie 1 reveal intact blood flow and the expected probing behavior of most resident macrophages in the gut. Scale bar = 60 µm.

In order to perform intravital microscopy of the villi in the small intestine, we developed a technique which allows multiphoton imaging from the luminal side of the gut in anesthetized mice, described in detail in Materials and Methods. In brief, an intestinal loop of the duodenum or proximal jejunum was carefully exteriorized and the lumen was opened with an incision on the antimesenteric line. The gut was mounted flat on a stage heated to body temperature, cleaned and covered by agarose and PBS, to prevent the tissue from drying out (Figure 1a). Imaging was performed with an upright two-photon laser-scanning system equipped with a Ti:Sa laser and an optical parametric oscillator for excitation, and filters as well as photomultiplier tubes for the simultaneous detection of three different wavelength ranges . In addition, the system contained a trigger box and a time-correlated single photon counter (TCSPC) device for fluorescence lifetime imaging (FLIM) (Figure 1b).

With this technique, intravital microscopy could be performed under physiologic conditions, as demonstrated by time lapse imaging (Figure 1c and Supplementary Movie 1). Intact blood flow was demonstrated in mice that were injected intravenously with rhodamine dextran before imaging. Non-migratory CX3CR1-GFP^+^ myeloid cells constantly sampled their surroundings by extending and retracting protrusions, and resembled the amoeboid-shaped myeloid lamina propria cells described previously [20]. By labeling the epithelium using an injection of the blue dye CMAC, we could observe myeloid cells sampling the epithelium by touching the bases of epithelial cells with their protrusions, and even reaching in between epithelial cells. When cell nuclei were labeled by injection of the nuclear dye Hoechst 33342, we could visualize the highly ordered cellular organization of the epithelial cells lining the villi, and motility was observed in a fraction of myeloid cells in the lamina propria, as described previously [30].

### 2.2. NAD(P)H Metabolism in Myeloid Cells of the Small Intestine under Steady State Conditions

**Figure 2:**
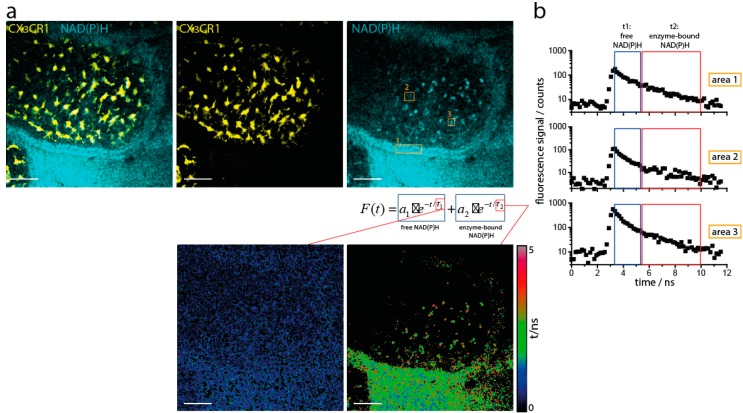

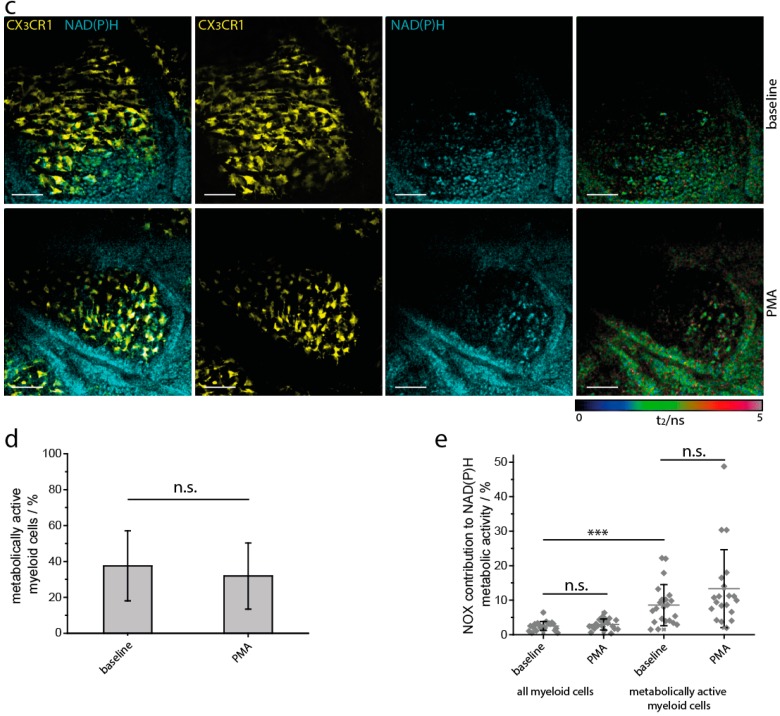
Intravital marker-free fluorescence lifetime imaging (FLIM) reveals NAD(P)H enzymatic activity in the myeloid compartment of the small intestine. (**a**) The upper row show merged and single fluorescence images of an intestinal villus in a CX3CR1-GFP mouse: cyan represents NAD(P)H fluorescence, yellow represents eGFP fluorescence. In each pixel of the image, the NAD(P)H fluorescence signal was acquired in a time-resolved manner and approximated by a bi-exponential function. In this way, the fluorescence lifetimes of the free and of the enzyme-bound NAD(P)H were calculated for each pixel and transformed into a free NAD(P)H fluorescence lifetime image (lower row, left, short fluorescence lifetimes) and an enzyme-bound NAD(P)H fluorescence lifetime image (lower row, right, long fluorescence lifetimes). Scale bar = 50 µm. (**b**) Typical NAD(P)H fluorescence decay curves in different regions of the villus: area 1 shows the NAD(P)H fluorescence signal of the epithelium, area 2 shows the dim NAD(P)H fluorescence signal of a myeloid cell; area 3 shows the bright NAD(P)H fluorescence signal of a myeloid cell. Scale bar = 50 µm. (**c**) Fluorescence intensity images of intestinal villi under steady-state conditions and upon phorbol myristate acetate (PMA) stimulation in CX3CR1-GFP mice: cyan represents NAD(P) fluorescence, yellow represents GFP fluorescence. Corresponding fluorescence lifetime images of enzyme-bound NAD(P)H, pseudocolored by lifetime. The 100 µM PMA solution was added locally at the imaging site and imaging was performed 30 min after treatment. Scale bar = 50 µm. (**d**) Overall NAD(P)H enzymatic activity in the myeloid compartment of the small intestine under steady-state conditions and upon PMA stimulation, as measured by NAD(P)H FLIM. (**e**) NOX enzymes contribution to the overall NAD(P)H enzymatic activity in myeloid cells under steady-state conditions and upon PMA treatment, as it results from NAD(P)H FLIM measurements. The graphs (**d**,**e**) display results acquired in *n* = 10 mice, 3–6 investigated areas per mouse. All mice were analyzed both in steady-state and after local treatment with PMA. Analysis in (**d**,**e**) was performed using one-way ANOVA statistical tests (*** *p* < 0.001).

The metabolism of myeloid cells generally underlays their various functions in the small intestine. Therefore, we analyzed their NAD(P)H metabolic activity under steady state conditions by means of in vivo NAD(P)H fluorescence lifetime imaging (Figure 2a). Based on the fluorescence decay curves of NAD(P)H in each pixel of the image (Figure 2b), we mapped the fluorescence lifetime of free NAD(P)H (approx. 400 ps), the fluorescence lifetime of enzyme-bound NAD(P)H, i.e., NAD(P)H involved in cellular processes, and the ratio between free and enzyme-bound NAD(P)H representing the NAD(P)H metabolic activity. While the fluorescence lifetime of free NAD(P)H can easily be measured in all cells of the intestinal tissue (Figure 2a, left image, lower panel), the fluorescence lifetime of enzyme-bound NAD(P)H can be detected only in certain tissue areas of the same region (Figure 2a, right image, lower panel). The areas in which enzyme-bound NAD(P)H could not be detected appear black in the fluorescence lifetime map of enzyme-bound NAD(P)H and indicate resting cells, with generally low metabolic activity. By using the simultaneously acquired fluorescent protein signal in reporter mice (either CX3CR1-GFP [20] or CD11c:tdRFP [31,32]), we identified in the NAD(P)H FLIM images the position of myeloid cells and quantified their metabolism (Figure 2c).

The overall activity of NAD(P)H-dependent enzymes as defined by NAD(P)H binding is rather low, approx. 40% (Figure 2d). This indicates that most of the resident myeloid cells are resting in terms of NAD(P)H-dependent metabolic activity. Even strong stimulation inducing an oxidative burst, the cell-permeable protein kinase C agonist phorbol myristate acetate (PMA), did not change the ratio between free and enzyme-bound NAD(P)H (Figure 2d). Despite their low metabolic activity, most of the resident macrophages appeared highly active in probing their environment (Supplementary Movie 1). Only a small fraction of the myeloid cells, especially localized at the villi tip, are metabolically active. We hypothesize that these cells fulfill a different function in the gut and further focused on phagocytosis as one of the main functions of myeloid cells.

During phagocytosis, the main NAD(P)H-dependent enzymes responsible for reactive oxygen species (ROS) production belong to the NOX family (NOX1-4, DUOX1,2). NOX2 is known to be highly expressed in myeloid cells [33]. We therefore focused on analyzing NOX activity by NAD(P)H-FLIM, since we have previously showed that NADPH bound to NOX enzymes displays a longer fluorescence lifetime of NADH and NADPH bound to typically active enzymes, i.e., 3600 ps as compared to approx. 1500–2000 ps [29]. We quantified the NOX activity contribution to the overall NAD(P)H-dependent enzymatic activity by calculating the ratio between the image area displaying NAD(P)H fluorescence lifetime values in the range 3300–3900 ps and the total area of myeloid cells [26,27,28,34]. As expected from the previous results, the general NOX activity was low when the whole myeloid population was investigated. However, when only metabolically active cells (defined by >10% bound NAD(P)H of the total NAD(P)H fluorescence signal, per CX3CR1+ area) were included in the analysis, NOX activity contribution amounted to 8.6 ± 2.9% (s.d.) (Figure 2e). In some of the analyzed regions of the villi, NOX activity contribution reached up to 25%, indicating that this phagocytic enzyme function can consume on average up to a quarter of the cellular coenzyme availability (Figure 2a,c). Notably, these regions were preferentially located at the tip of the villi. Next, we added PMA to the tissue for determination of maximal NOX activation capacity. In cells with low metabolic activity, even this strong stimulus did not induce an increase, neither in NOX, nor in general NAD(P)H-dependent enzymatic activity (Figure 2d,e). In metabolically active cells, NOX activation could not be significantly increased by PMA, implying that these cells had already reached their maximum oxidative burst capacity in the steady state.

### 2.3. NOX Activity is Present in Epithelial Cells of the Small Intestine in Steady State

**Figure 3:**
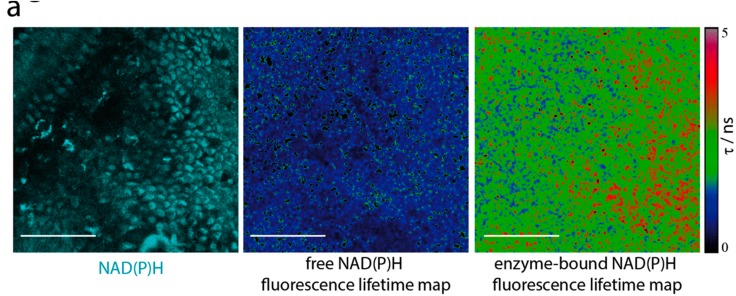

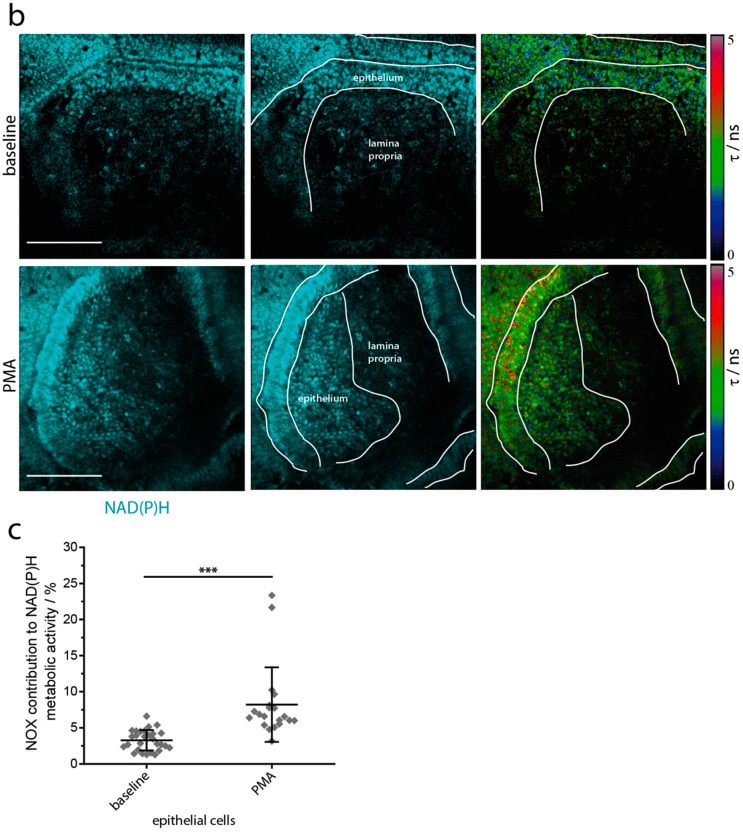
Intravital marker-free FLIM reveals sustained NAD(P)H oxidase (NOX) activity in the epithelium of intestinal villi. (**a**) NAD(P)H fluorescence intensity (cyan), fluorescence lifetime image of free NAD(P)H and fluorescence lifetime images of enzyme-bound NAD(P)H of the highly ordered epithelium of an intestinal villus of a mouse. In the entire epithelial compartment, in this case comprising the entire displayed tissue area, not only free but also enzyme-bound NAD(P)H signal was detected indicating its high metabolic activity. Scale bar = 100 µm. (**b**) Fluorescence intensity and enzyme-bound NAD(P)H fluorescence lifetime images of intestinal villi comprising both epithelial and lamina propria regions under steady-state conditions and upon PMA stimulation. Scale bar = 100 µm. (**c**) NOX enzymes contribution to the overall NAD(P)H enzymatic activity in epithelial cells under steady-state conditions and upon PMA treatment, generated from NAD(P)H FLIM measurements depicted in (**b**). The graph (**c**) displays results acquired in *n* = 10 mice, 3–6 investigated areas per mouse—the same mice as in Figure 2. Analysis in (**c**) was performed using one-way ANOVA statistical test (*** *p* < 0.001).

While analyzing the NAD(P)H metabolic activity in myeloid cells of the villi, we noticed an elevated NOX activity in epithelial regions as well (Figure 2a,b; Area 1). This led us to investigate the NAD(P)H-dependent metabolism of epithelial cells in more depth. In contrast to the myeloid cell population in the lamina propria, the epithelial cells showed a high NAD(P)H-dependent enzymatic activity (Figure 3a). This included NOX enzymes, which took up around 5% of the total NAD(P)H-dependent enzymatic activity in epithelial regions (Figure 3b,c). Addition of PMA led to a significant increase in NOX activity in the epithelium (Figure 3c).

### 2.4. NOX Isotype Expression in Various Cellular Compartments of the Small Intestine

**Figure 4:**
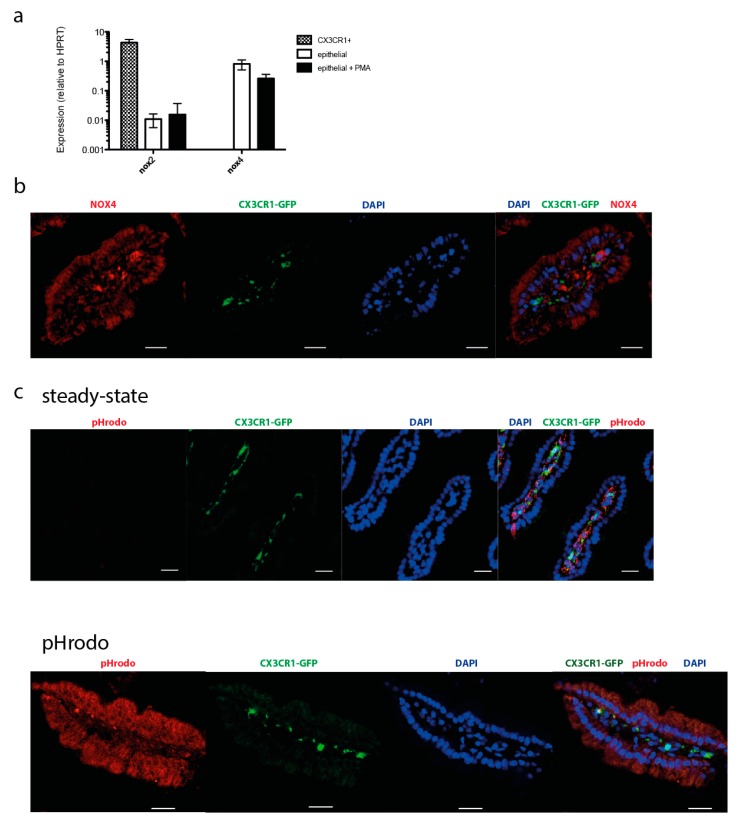
Expression of NOX isotypes in the epithelial and myeloid compartments of the small intestine. (**a**) RT-PCR. CX3CR1-GFP^+^ and epithelial cells were sorted from the small intestine to examine their expression of NOX enzymes in the steady-state or after activation with PMA. Bar graph indicates the relative expression of Nox2 and Nox4 relative to HPRT. No significant difference was observed upon PMA treatment. Results shown are representative of three independent experiments; columns and error bars indicate mean +/− SEM. (**b**) Immunofluorescence micrograph of the small intestine from a CX3CR1-GFP mouse, immunostained for Nox4. Nox4 is expressed at high levels in GFP^+^ and GFP^−^ cells in the LP, and at moderate levels in epithelial cells. Scale bar is 20 µm. (**c**) Immunofluorescence micrograph of the small intestine before (top) and after (bottom) incubation with pHrodo bioparticles. At physiologic pH the pHrodo beads are non-fluorescent. pHrodo accumulation is seen as bright clusters in GFP^+^ and GFP^−^ cells in the LP, and diffusely throughout the epithelium. In the steady-state, no pHrodo signal is observed. pHrodo bioparticles are rapidly taken up and found in acidic compartments of GFP^+^ and GFP^−^ cells. Immunofluorescence images are representative of three independent experiments; scale bar is 20 µm.

Our experiments reveal NAD(P)H FLIM as a versatile tool to analyze pan-NOX activity in the small intestine in vivo. In order to obtain information on the specific NOX isotypes present in the various cellular compartments where enzymatic NOX activity was observed, we analyzed the expression of NOX family members. It is generally accepted that NOX2 is the most abundant isoform in myeloid cells [33], and it has been shown previously that NOX4 is expressed in gut epithelium [35]. We therefore focused on the analysis of these two isotypes. PCR analysis confirmed a high expression of NOX2 in sorted CX3CR1^+^ cells from the small intestine while NOX4 was undetectable (Figure 4a). Neither NOX2 nor NOX4 expression showed significant changes upon 30 min stimulation with PMA (Figure 4a). Enterocytes expressed NOX4 at high levels and had markedly lower levels of NOX2. The expression of NOX4 could also be confirmed by histological analysis, showing localization of NOX4 in epithelial cells (Figure 4b).

To determine if pHrodo bioparticles are taken up by CX3CR1-GFP^+^ cells, epithelial cells, or other cells, we applied pHrodo bioparticles to the intestine, and examined sections by immunofluorescence. At physiologic pH the pHrodo beads are non-fluorescent; upon acidification in endo-/lysosomes the particles become fluorescent. In the steady-state, no pHrodo signal is observed (Figure 4c, top). Upon stimulation with pHrodo, we observed significant colocalization of pHrodo particles within the acidic compartments of GFP^+^ and GFP^−^ cells, and a more diffuse staining throughout the epithelial cells (Figure 4c, bottom).

**Figure 5:**
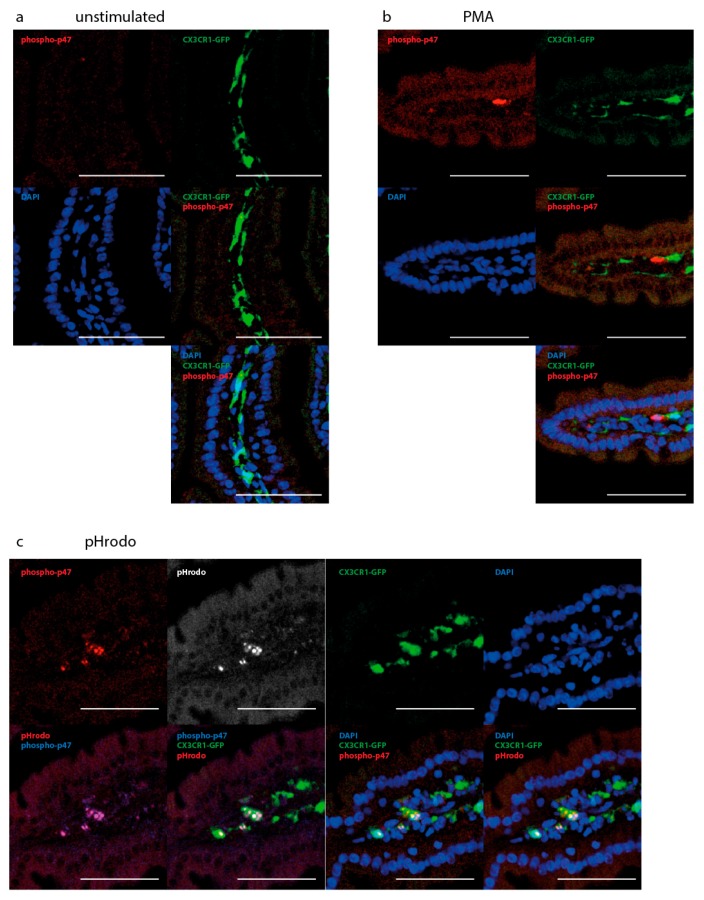
Induction of p47phox-phosphorylation by activating stimuli. (**a**) Immunofluorescence micrograph of the small intestine from a CX3CR1-GFP mouse, immunostained for phospho-p47. In the steady-state, minimal p47 phosphorylation is observed. (**b**) Immunofluorescence micrograph of the small intestine from a CX3CR1-GFP mouse, after incubation with PMA for 10 min, immunostained for phospho-p47. p47Phox is rapidly phosphorylated at Ser359 upon PMA stimulation in epithelial and LP cells. (**c**) Immunofluorescence micrograph of the small intestine from a CX3CR1-GFP mouse, after application of pHrodo bioparticles for 30 min, pHrodo particles colocalize with phospho-p47, and phospho-p47 is largely restricted to pHrodo^+^ GFP^+^ cells. Epithelial cells exhibit a low level of p47 phosphorylation after incubation with pHrodo particles, but the increase is not as marked as with PMA stimulation. Immunofluorescence images are representative of two independent experiments, Scale bar: 50 µm.

To further validate that PMA and pHrodo bioparticles activate enterocytes and myeloid cells, we examined p47 phosphorylation. In response to activating stimuli, p47 as a part of the NOX enzyme complex is phosphorylated, which allows binding to p22 and translocation to the membrane. We applied PMA or pHrodo bioparticles to the intestine, and examined sections by immunofluorescence for p47 phosphorylation (Figure 5). We observed a very low level of background p47 phosphorylation (Figure 5a). Upon application of PMA or pHrodo bioparticles, p47 phosphorylation was rapidly induced, however, the cellular compartments where it occurred differed between both stimuli: PMA induced p47 phosphorylation in epithelial cells as well as in GFP^+^ and GFP^−^ LP cells (Figure 5b). pHrodo particles induced a strong p47 phosphorylation signal, mostly overlapping with the pHrodo^+^ compartments in the GFP^+^ myeloid cells. A fainter, diffuse signal for p47 phosphorylation was detected in the epithelial cells (Figure 5b,c).

### 2.5. NAD(P)H-Metabolism in the Small Intestine in Response to Inflammatory Stimuli

The gut surface is continually exposed to a large number of microorganisms from birth until death. In healthy individuals, several components of the intestinal immune system work together to ensure a fine-tuned balance of pro- and anti-inflammatory signals in response to those microorganisms. ROS generated by NOX enzymes are known to play an important role in host defense [33,36]. Therefore, we next addressed the question whether inflammatory stimuli from pathogenic bacteria can induce NOX activation in various compartments of the gut.

**Figure 6:**
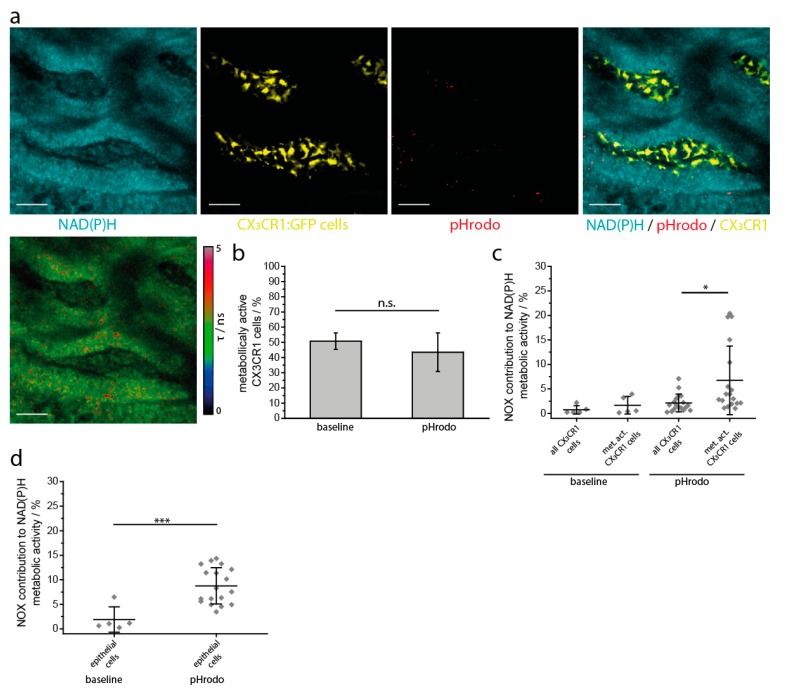
Intravital marker-free FLIM reveals compartmentalization of NOX enzymatic activity in the small intestine upon inflammatory stimulation. (**a**) The upper row shows single and merged fluorescence images of intestinal villi in a CX3CR1-GFP mouse locally treated with *S. aureus* pHrodo beads: cyan represents NAD(P)H fluorescence, yellow represents GFP fluorescence, red represents phagocytosed pHrodo beads in a low pH environment. At physiologic pH the pHrodo beads are non-fluorescent. A volume of 100 µL with 10 [8] pHrodo beads was added directly to the exposed inner surface of the small intestine. The image in the bottom row represents the corresponding fluorescence lifetime image of enzyme-bound NAD(P)H of the entire tissue. Scale bar = 50 µm. (**b**) Overall NAD(P)H enzymatic activity in the myeloid compartment of the small intestine under steady-state conditions and upon *S. aureus* pHrodo stimulation, as measured by NAD(P)H FLIM. (**c**) NOX enzymes contribution to the overall NAD(P)H enzymatic activity in myeloid cells under steady-state conditions and upon *S. aureus* pHrodo treatment, as calculated from NAD(P)H FLIM measurements. (**d**) NOX enzymes contribution to the overall NAD(P)H enzymatic activity in the epithelial compartment under steady-state conditions and upon *S. aureus* pHrodo treatment. The graphs (**b**–**d**) display results acquired in *n* = 5 mice, 1 baseline area and 3–5 areas after treatment in each mouse. Analysis in (**d**) was performed using one-way ANOVA statistical tests (* *p* < 0.05; *** *p* < 0.001, n.s.: not significant).

In order to mimic such stimuli, we added phagocytic beads coated with immunogenic membrane components of *S. aureus* (pHrodo) and investigated the NAD(P)H-dependent enzymatic activity in the gut by NAD(P)H-FLIM (Figure 6a). Similar to our previous observations under steady state conditions, pHrodo addition did not increase the overall NAD(P)H metabolic activity of the myeloid cells (Figure 6b). Similar to baseline and PMA treatment, NOX activity was significantly increased when metabolically active myeloid cells were analyzed (Figure 6c). Notably, in epithelial cells, pHrodo led to a ~5-fold increase in mean NOX activity within 30 min (Figure 6d). This increase in activity did not coincide with any change in general metabolic activity (defined by the ratio between free and enzyme bound NAD(P)H) of epithelial cells upon pHrodo treatment (data not shown), indicating that NOX activation occurs at the expense of other NAD(P)H-dependent enzymes. When the area of NOX activity resulting from NAD(P)H-FLIM measurements was analyzed with respect to its cellular sources, the main contributor after *S. aureus* pHrodo treatment was the epithelial compartment (69 ± 10%), whereas the CX_3_CR1^+^ myeloid cells played only a marginal role (3.0 ± 1.2%).

## 3. Discussion

Members of the NOX family of enzymes (NOX1-4, DUOX1,2) are expressed in many different cell types throughout the body [37]. The main function of these NAD(P)H-dependent enzymes is the generation of reactive oxygen species (ROS), which are released from the cells into their surroundings and play crucial roles in host defense [37]. Cells of the innate immune system, especially neutrophils and macrophages, are known to express high levels of NOX enzymes, with NOX2 being the most prevalent isotype in these phagocytic cells [33,38]. The NOX enzyme complex consists of two membrane bound subunits (gp91-phox and p22-phox), the cytosolic subunits p47-phox, p67-phox and p40-phox, as well as the G-protein Rac. Phosphorylation of p47-phox during NOX activation results in re-localization of the cytosolic subunits to the membrane, where SH3 domains of proline–rich regions of p47 interact with SH3 domains of p22. Ultimately, this results in the production of superoxide, by reducing molecular oxygen with the help of NAD(P)H as coenzyme. Importantly, the fluorescence lifetime differs between free and enzyme-bound NAD(P)H, and this phenomenon can be used to analyze the overall metabolic activity in tissues, by determining the amount of free (fluorescence lifetime approx. 400 ps) versus enzyme-bound (fluorescence lifetime more than 1000 ps) NAD(P)H. Moreover, by taking advantage of the very distinct, long fluorescence lifetime of NOX (3600 ps), we can specifically image and quantify NOX activation in those tissues. Here, we for the first time apply this technique to analyze NAD(P)H–dependent enzyme activity in various cellular compartments of the small intestine in living mice. By combining TCSPC detection with PMT-based spectral detection, we are able to not only determine the overall metabolic activity in the tissue, but also trace this activity back to its cellular origin in order to identify the cellular source of activated NOX enzymes.

Using this method, we found NOX activation in CX3CR1^hi^ cells by intravital imaging of the small intestine in mice. Interestingly, we could not elicit a sustained and long-term NOX activation in the majority of those cells; rather, they were metabolically resting and only 40% exhibited overall NAD(P)H-dependent metabolic activity, as defined by total enzyme—bound NAD(P)H. In addition, we noted also regional differences of the CX3CR1^hi^ macrophages, as NOX and general metabolic activity were both mainly observed at the tip of the villi, indicating a compartmentalization or functional diversification within the resident CX3CR1^hi^ cell population. Despite their metabolically resting state, the CX3CR1^hi^ cells were actively probing their surroundings, as demonstrated by protrusions which they constantly moved, presumably sampling their environment.

Even after activation with PMA, an extremely strong stimulus, their increase in capacity to activate NOX was relatively small. Seemingly in contrast to our findings, NOX1 expressed in macrophages has been shown to play a role in the pathogenesis of colitis in a mouse model [39] and NOX2-derived ROS was demonstrated to be involved in the development of necrotizing enterocolitis [40]. However, it should be noted that the presence of ROS in the tissues was analyzed in those publications, whereas our focus here was on determining the activation level of NOX, the main enzyme family responsible for the production of extracellular ROS. More importantly, we did not analyze intestinal cells under conditions of prolonged active inflammation, as was the case in these two reports.

Consistent with our findings, resident CX3CR1^hi^ cells in the healthy intestine have been found to be important for tissue homeostasis by tissue clearance [41,42,43]. Moreover, CX3CR1^hi^ macrophages do not express co-stimulatory molecules such as CD80, CD86 or CD40, and are inert to inflammatory stimuli. Although intestinal macrophages express a wide range of TLRs, they do not respond to TLR activation by producing pro-inflammatory mediators, as macrophages in other tissues do [17,41,42] and they do not generate nitric oxide or reactive oxygen species during phagocytosis [44,45]. However, they constitutively produce TNFα and express high levels of MHCII [22]. Although they take up luminal antigens and microorganisms, they are non-migratory and do not translocate to mesenteric lymph nodes following antigen-uptake [18], therefore they are unlikely to be involved in the initial priming of naïve T cells. In contrast, they possess anti-inflammatory properties, mediated in part by constitutive IL-10 production [24,46]. This makes them essential for oral tolerance, as CX3CR1-deficient mice fail to recruit to and maintain Treg cells in the LP [47]. It has recently been shown that the anti-inflammatory signature of CX3CR1^+^ macrophages critically depends on their expression of the IL-10-receptor (IL-10R), and that they gain anti-inflammatory properties via IL-10 from Foxp3^+^ Treg cells [48]. The same study showed that the knock-out of IL-10R in CX3CR1^+^ macrophages leads to spontaneous enterocolitis.

Taken together, our results fit into the emerging picture of CX3CR1^+^ macrophages having mainly anti-inflammatory properties and add an intrinsic limitation of NOX activation capacity as another mechanism by which the anti-inflammatory properties of these cells are maintained. In contrast, during inflammatory conditions such as colitis, Ly6C^+^ blood monocytes accumulate in a CCR2-dependent manner in the gut lamina propria and locally differentiate further into Ly6C^+^CX3CR1^int^ monocytes [49,50]. These monocytes show a pro-inflammatory phenotype during colitis and ablation of these cells leads to amelioration of disease [23]. In contrast, resident macrophages have not only been shown to play a role in tissue homeostasis in the steady state, but in promoting resolution of inflammation [51]. In future studies, it will be important to compare the NOX activation capacity between inflammatory monocytes and the resident CX3CR1^hi^ macrophages, in order to further define functional differences between these cell types in mouse models of intestinal inflammation.

In addition to myeloid cells, we discovered that enterocytes show a significant amount of NOX activity in vivo. Their contribution to the total tissue NOX activity was actually ~20× higher than that of CX_3_CR1^+^ cells, making them the largest cellular compartment with NOX activity in the gut. We found NOX4 to be the main NOX family member expressed in intestinal epithelial cells, consistent with a report by Hornef and colleagues [35]. Rather than acting bactericidally, NOX-derived ROS from enterocytes were suggested by these authors to act as signaling mediators in horizontal cell-cell communication. Thereby, a coordinated immune response of the epithelium in response to infection with bacterial pathogens is induced. This inducible immune response of enterocytes is consistent with their ability to present MHCII:peptide complexes [52,53,54] and cytokines under conditions of inflammation [55].

Our data on the expression of NOX2 in myeloid cells, as well as on NOX4 in epithelial cells in the intestine, and the activation kinetics of NOX measured by FLIM, support the hypothesis that ROS production in response to a pathogen occurs within the time frame of minutes, therefore transcriptional regulation does not play a role in these first lines of defense. Rather, the process is regulated at the post-translational level of NOX activation, which underlines the importance of in vivo analysis by FLIM. Whether epithelial-derived ROS is mainly acting as a signaling mediator or has bactericidal functions remains to be analyzed in the future. By immunofluorescence, the subcellular distribution pattern of phosphorylated p47 clearly differs in the two cell types we investigated here: in myeloid cells it localized to sub-cellular compartments, likely representing phagosomes as indicated by its colocalization with phagocytosed pHrodo particles, while epithelial cells displayed a more diffuse staining pattern throughout the cytoplasm, with no polarity in the cell or accumulation within certain cellular compartments. Extending NAD(P)H-FLIM analysis over longer time periods may help to elucidate the spatiotemporal sequence of NOX activation in different cellular and sub-cellular compartments in order to address those questions.

Notably, besides their beneficial role in host defense, NOX activation can also have detrimental effects, for example during neuroinflammation, where NOX-overactivation in myeloid cells results in excessive ROS generation and subsequent neuronal dysfunction and damage, as shown by us and others [26]. During chronic inflammation, both leukocytes and non-hematopoietic, tissue-resident cells been shown to over-activate NOX, thereby contributing to the perpetuation of local inflammation [27,28,56]. It will be important to determine whether similar cellular and molecular mechanisms apply to chronic inflammation in the intestine. Of note, the label-free nature of NAD(P)H-FLIM makes it a tool for studying these mechanisms not only in mice, but also for potentially translational studies involving human tissues.

## 4. Materials and Methods

### 4.1. Multi-Photon Microscope Setup for Fluorescence Lifetime Imaging (FLIM)

Experiments were performed using a specialized multi-photon laser-scanning microscope for fluorescence lifetime imaging (FLIM) as previously reported [26,57]. In brief, the beam of a tunable fs-pulsed Ti:Sa laser (wavelength range 700–1080 nm, 140 fs, 80 MHz, Chameleon Ultra II, Coherent, Dieburg, Germany) is scanned by two galvanometric mirrors and focused into the sample by an objective lens for deep-tissue imaging (20× dipping lens, NA 0.95, WD 2 mm—Olympus, Hamburg, Germany or 20× dipping lens, NA 1.00, WD 1.2 mm—Zeiss AG, Jena, Germany) [26,57]. The resulting fluorescence signal is detected and analyzed either by a time-correlated single-photon counting (TCSPC) point detector (FLIM TCSPC, LaVision Biotec GmbH, Bielefeld, Germany) or photomultiplier tubes (Hamamatsu, Japan). The TCSPC device is based on photon detection with a hybrid photomultiplier tube and on evaluation relying on time-to-digital converter (TDC) electronics. The electronic dead time of the device is less than 5 ns. Spectral discrimination of the fluorescence signal was achieved by appropriate dichroic mirrors and interference filters. NADH and NADPH (together NAD(P)H) were excited at 760 nm and detected through an interference filter at 460 ± 30 nm. The time step (bin) was 27 ps and the time window for measuring the fluorescence decay was 9 ns. EGFP in the CX3CR1^hi^ resident macrophages [20] and rhodamine dextran were simultaneously excited at 850 nm and detected through a dichroic mirror (cut off wavelength 560 nm) and interference filters of 525 ± 25 nm and 593 ± 20 nm, respectively. When *CD11c:Cre × Rosa26-flox-STOP-flox-tdRFP* mice were used in intravital experiments of the small intestine, we employed infrared excitation of an optical parametric oscillator (OPO, APE, Berlin, Germany) tuned to 1110 nm.

### 4.2. Mice

All mice used were on a C57/Bl6J background. The *CX3CR1-GFP^+/−^* mouse [20] was used to detect resident macrophages within the gut and rhodamine dextran was i.v. injected to label blood flow in vivo. *CD11c:Cre × Rosa26-flox-STOP-flox-tdRFP* [31,32] mice were imaged to identify myeloid cells in the small intestine. Red fluorescence in myeloid cells in the intestine was confirmed by flow cytometry and the cells exhibited the characteristic myeloid morphology similar toCX3CR1^hi^ cells within the lamina propria. All animal experiments were approved by the Landesamt für Gesundheit und Soziales, Berlin, Germany in accordance with institutional, state and federal guidelines.

### 4.3. Intravital Imaging of the Small Intestine

We developed a specialized imaging technique approaching the gut from the luminal side. Thereby, mice were anesthetized and placed in the lateral decubitus position on a custom-built imaging-stage kept constantly at 37 °C. Additionally, a temperature sensor was immobilized on top of the heated stage underneath the envisaged imaging area to monitor the tissue temperature at the imaging site. A 1 cm incision was made in the abdominal skin and the muscular wall along the median line under semi-sterile conditions and an intestinal loop of the duodenum or proximal jejunum was carefully exteriorized. Care was taken to preserve the integrity of blood and lymph vessels. The exteriorized intestinal loop was then fixed on top of the heated stage above the temperature sensor using tissue adhesive. The intestinal tube was cut along the anti-mesenteric line and the upper part was folded back. The cutting edges were cauterized to prevent bleeding and the intestinal contents were gently flushed away with PBS and wiped to obtain a clean surface. The imaging area was surrounded with 1% agarose gel to form a basin which was filled with PBS to prevent the tissue from drying out. The imaging field was covered with one drop of agarose gel and a cover glass to fix the intestinal villi in a lateral position, enabling the imaging of an entire villus in one image stack. In some experiments, we labeled nuclei in vivo by i.v. injection of Hoechst (8 mg/kg) or labeled epithelial cells by local application of 20 µL of CellTracker Blue (7-Amino-4-Chlormethylcoumarin, CMAC) at a concentration of 30 µM, directly onto the tissue immediately before imaging. It should be noted that both of these dyes emit in the blue region and will thereby preclude further NAD(P)H FLIM measurements.

### 4.4. In Vitro Activation

Animals were sacrificed by cervical dislocation, and the proximal small intestines were harvested, flushed of feces, and incubated in RPMI with 10% serum in the presence or absence of PMA (100 µM) or pHrodo Red bioparticles (0.1 mg/mL). For histological examination, intestinal segments were fixed in paraformaldehyde overnight, cryopreserved in 30% sucrose, embedded in OCT, frozen on dry ice, and cut into 7 µm sections on a cryotome (Leica, Wetzlar, Germany). For cell sorting, single-cell suspensions were prepared by a modification of the procedure of Farache et al. [58]. Briefly, intestinal segments were opened longitudinally, cut into 0.5 cm sections, and digested in a mixture of collagenase VIII and collagenase D in RPMI with 10% serum for 25 min, until the fragments were dissociated. Cells were filtered through 100 µm and 70 µm filters, centrifuged, and stained with antibodies.

### 4.5. Cell Sorting

Single-cell suspensions prepared as above were incubated with Fc-block (anti-CD16/CD32 clone 2.4G2, produced in-house) to minimize nonspecific FcR-mediated labeling. Cells were stained with anti-EpCAM antibodies (BioLegend, Koblenz, Germany) to identify epithelial cells, anti-CD45 antibodies (BioLegend, Koblenz, Germany) to identify hematopoietic cells, and CX3CR1-GFP was sorted based on endogenous GFP fluorescence. DAPI (Sigma-Aldrich, Munich, Germany) was added shortly prior to sorting for dead cell exclusion. Cells were sorted on a FACSAria (Becton Dickinson, Heidelberg, Germany) into cold RPMI with 10% FCS [59], and immediately following cessation of the sort, RNA was isolated from the cells. From a typical sort, 15,000 macrophages and 500,000 epithelial cells were isolated.

### 4.6. RT-PCR

RNA was isolated from cells using the Promega SV Total RNA Isolation kit, according to the manufacturer’s protocol. Following elution, RNA was aliquoted and stored at −80 °C. Reverse transcription was performed with the Applied Biosystems high capacity RNA-to-cDNA kit, according to the manufacturer’s protocol, and cDNA was stored at −20 °C. Real-time PCR was performed using TaqMan reagents: TaqMan Universal PCR Master Mix (Applied Biosystems/ThermoFisher Scientific, Waltham, MA, USA), and target gene assay mix containing sequence-specific primers and 6-carboxyfluorescein (6-FAM) dye–labeled TaqMan minor groove binder (MGB) probe (Applied Biosystems, Waltham, MA, USA) were used. Sequence-specific probes were Nox2 (assay ID Mm01287743_m1), Nox4 (assay ID Mm00479246_m1), and HPRT was used as the endogenous reference for normalization (assay ID Mm03024075_m1). Samples were run in triplicate on a StepOnePlus (Applied Biosystems, Waltham, MA, USA) using the same quantity of cDNA for each sample, and expression was calculated using the ΔCT method and plotted as relative expression ((delta CT)^2^).

### 4.7. Immunofluorescence

Frozen sections were thawed, rehydrated in PBS, blocked with serum and Fc-block, and immunostained for Nox4 (rabbit polyclonal, Novus Biologicals, Littleton, CO, USA), p47 (goat polyclonal, Abcam, Cambridge, UK), or phospho-p47 Ser359 (rabbit polyclonal, Abcam, Cambridge, UK). Samples were incubated with primary antibodies for 60 min, washed 4 times in PBS with 0.1% Tween-20, and stained with the appropriate Alexa 647-labeled secondary antibody (donkey anti-goat or anti-rabbit). Following labeling with secondary antibodies, slides were washed and mounted in Fluoromount-G with DAPI. Samples were examined on a Zeiss LSM710, with the appropriate laser and filter settings. pHrodo was visualized by its endogenous fluorescence, and GFP was detected either by its endogenous fluorescence or with anti-GFP antibodies (Rockland, Limerick, PA, USA) labeled in-house with Alexa 488.

## Supplementary Materials

Supplementary Movie 1 can be found at http://www.mdpi.com/1422-0067/19/5/1365/s1.

## Abbreviations

EGFP	Enhanced green fluorescent protein
FLIM	Fluorescence lifetime imaging
GI	Gastro-intestinal
IEL	intraepithelial lymphocytes
LP	lamina propria
MHC	Major histocompatibility complex
NAD(P)H	Nicotinamide Adenine Dinucleotide (Phosphate) Dehydrogenase
NOX	NADPH oxidases
PMA	Phorbol myristate acetate
ROS	Reactive oxygen species (ROS)
TCSPC	Time-correlated single photon counter
Ti:Sa	Titanium-sapphire
TLR	Toll-like receptor

## References

[1] Mowat A.M. (2003). Anatomical basis of tolerance and immunity to intestinal antigens. Nat. Rev. Immunol..

[2] Peterson L.W., Artis D. (2014). Intestinal epithelial cells: Regulators of barrier function and immune homeostasis. Nat. Rev. Immunol..

[3] Lemke A., Kraft M., Roth K., Riedel R., Lammerding D., Hauser A.E. (2016). Long-lived plasma cells are generated in mucosal immune responses and contribute to the bone marrow plasma cell pool in mice. Mucosal Immunol..

[4] He B., Xu W., Santini P.A., Polydorides A.D., Chiu A., Estrella J., Shan M., Chadburn A., Villanacci V., Plebani A. (2007). Intestinal bacteria trigger t cell-independent immunoglobulin A_2_ class switching by inducing epithelial-cell secretion of the cytokine April. Immunity.

[5] Xu W., He B., Chiu A., Chadburn A., Shan M., Buldys M., Ding A., Knowles D.M., Santini P.A., Cerutti A. (2007). Epithelial cells trigger frontline immunoglobulin class switching through a pathway regulated by the inhibitor slpi. Nat. Immunol..

[6] Iliev I.D., Mileti E., Matteoli G., Chieppa M., Rescigno M. (2009). Intestinal epithelial cells promote colitis-protective regulatory t-cell differentiation through dendritic cell conditioning. Mucosal Immunol..

[7] Rimoldi M., Chieppa M., Salucci V., Avogadri F., Sonzogni A., Sampietro G.M., Nespoli A., Viale G., Allavena P., Rescigno M. (2005). Intestinal immune homeostasis is regulated by the crosstalk between epithelial cells and dendritic cells. Nat. Immunol..

[8] Taylor B.C., Zaph C., Troy A.E., Du Y., Guild K.J., Comeau M.R., Artis D. (2009). TSLP regulates intestinal immunity and inflammation in mouse models of helminth infection and colitis. J. Exp. Med..

[9] Goto Y., Ivanov I.I. (2013). Intestinal epithelial cells as mediators of the commensal-host immune crosstalk. Immunol. Cell Biol..

[10] Laky K., Lefrancois L., Lingenheld E.G., Ishikawa H., Lewis J.M., Olson S., Suzuki K., Tigelaar R.E., Puddington L. (2000). Enterocyte expression of interleukin 7 induces development of gammadelta T cells and peyer’s patches. J. Exp. Med..

[11] Kennedy M.K., Glaccum M., Brown S.N., Butz E.A., Viney J.L., Embers M., Matsuki N., Charrier K., Sedger L., Willis C.R. (2000). Reversible defects in natural killer and memory CD8 T cell lineages in interleukin 15-deficient mice. J. Exp. Med..

[12] Cepek K.L., Shaw S.K., Parker C.M., Russell G.J., Morrow J.S., Rimm D.L., Brenner M.B. (1994). Adhesion between epithelial cells and T lymphocytes mediated by e-cadherin and the alpha e beta 7 integrin. Nature.

[13] Leishman A.J., Naidenko O.V., Attinger A., Koning F., Lena C.J., Xiong Y., Chang H.C., Reinherz E., Kronenberg M., Cheroutre H. (2001). T cell responses modulated through interaction between cd8alphaalpha and the nonclassical mhc class I molecule, TL. Science.

[14] Hume D.A., Perry V.H., Gordon S. (1984). The mononuclear phagocyte system of the mouse defined by immunohistochemical localisation of antigen F4/80: Macrophages associated with epithelia. Anat. Rec..

[15] Pull S.L., Doherty J.M., Mills J.C., Gordon J.I., Stappenbeck T.S. (2005). Activated macrophages are an adaptive element of the colonic epithelial progenitor niche necessary for regenerative responses to injury. Proc. Natl. Acad. Sci. USA.

[16] Zigmond E., Jung S. (2013). Intestinal macrophages: Well educated exceptions from the rule. Trends Immunol..

[17] Bain C.C., Scott C.L., Uronen-Hansson H., Gudjonsson S., Jansson O., Grip O., Guilliams M., Malissen B., Agace W.W., Mowat A.M. (2013). Resident and pro-inflammatory macrophages in the colon represent alternative context-dependent fates of the same Ly6Chi monocyte precursors. Mucosal Immunol..

[18] Bain C.C., Mowat A.M. (2014). Macrophages in intestinal homeostasis and inflammation. Immunol. Rev..

[19] Bain C.C., Mowat A.M. (2011). Intestinal macrophages—Specialised adaptation to a unique environment. Eur. J. Immunol..

[20] Jung S., Aliberti J., Graemmel P., Sunshine M.J., Kreutzberg G.W., Sher A., Littman D.R. (2000). Analysis of fractalkine receptor CX_3_CR1 function by targeted deletion and green fluorescent protein reporter gene insertion. Mol. Cell. Biol..

[21] Bain C.C., Bravo-Blas A., Scott C.L., Perdiguero E.G., Geissmann F., Henri S., Malissen B., Osborne L.C., Artis D., Mowat A.M. (2014). Constant replenishment from circulating monocytes maintains the macrophage pool in the intestine of adult mice. Nat. Immunol..

[22] Bain C.C., Mowat A.M. (2014). The monocyte-macrophage axis in the intestine. Cell. Immunol..

[23] Zigmond E., Varol C., Farache J., Elmaliah E., Satpathy A.T., Friedlander G., Mack M., Shpigel N., Boneca I.G., Murphy K.M. (2012). Ly6c hi monocytes in the inflamed colon give rise to proinflammatory effector cells and migratory antigen-presenting cells. Immunity.

[24] Weber B., Saurer L., Schenk M., Dickgreber N., Mueller C. (2011). CX_3_CR1 defines functionally distinct intestinal mononuclear phagocyte subsets which maintain their respective functions during homeostatic and inflammatory conditions. Eur. J. Immunol..

[25] Niesner R., Narang P., Spiecker H., Andresen V., Gericke K.H., Gunzer M. (2008). Selective detection of NADPH oxidase in polymorphonuclear cells by means of nad(p)h-based fluorescence lifetime imaging. J. Biophys..

[26] Mossakowski A.A., Pohlan J., Bremer D., Lindquist R., Millward J.M., Bock M., Pollok K., Mothes R., Viohl L., Radbruch M. (2015). Tracking CNS and systemic sources of oxidative stress during the course of chronic neuroinflammation. Acta Neuropathol..

[27] Radbruch H., Bremer D., Guenther R., Cseresnyes Z., Lindquist R., Hauser A.E., Niesner R. (2016). Ongoing oxidative stress causes subclinical neuronal dysfunction in the recovery phase of EAE. Front. Immunol..

[28] Radbruch H., Mothes R., Bremer D., Seifert S., Kohler R., Pohlan J., Ostendorf L., Gunther R., Leben R., Stenzel W. (2017). Analyzing nicotinamide adenine dinucleotide phosphate oxidase activation in aging and vascular amyloid pathology. Front. Immunol..

[29] Lakowicz J.R., Szmacinski H., Nowaczyk K., Johnson M.L. (1992). Fluorescence lifetime imaging of free and protein-bound NADH. Proc. Natl. Acad. Sci. USA.

[30] Kriegel F.L., Kohler R., Bayat-Sarmadi J., Bayerl S., Hauser A.E., Niesner R., Luch A., Cseresnyes Z. (2018). Cell shape characterization and classification with discrete Fourier transforms and self-organizing maps. Cytometry A.

[31] Caton M.L., Smith-Raska M.R., Reizis B. (2007). Notch-RBP-J signaling controls the homeostasis of CD8- dendritic cells in the spleen. J. Exp. Med..

[32] Luche H., Weber O., Nageswara Rao T., Blum C., Fehling H.J. (2007). Faithful activation of an extra-bright red fluorescent protein in “knock-in” cre-reporter mice ideally suited for lineage tracing studies. Eur. J. Immunol..

[33] Babior B.M. (1999). NADPH oxidase: An update. Blood.

[34] Bremer D., Leben R., Mothes R., Radbruch H., Niesner R. (2017). Method to detect the cellular source of over-activated NADPH oxidases using NADPH fluorescence lifetime imaging. Curr. Protoc. Cytom..

[35] Dolowschiak T., Chassin C., Ben Mkaddem S., Fuchs T.M., Weiss S., Vandewalle A., Hornef M.W. (2010). Potentiation of epithelial innate host responses by intercellular communication. PLoS Pathog..

[36] Lambeth J.D., Neish A.S. (2014). Nox enzymes and new thinking on reactive oxygen: A double-edged sword revisited. Annu. Rev. Pathol..

[37] Bedard K., Krause K.H. (2007). The NOX family of ROS-generating NADPH oxidases: Physiology and pathophysiology. Physiol. Rev..

[38] Babior B.M. (2000). Phagocytes and oxidative stress. Am. J. Med..

[39] Yokota H., Tsuzuki A., Shimada Y., Imai A., Utsumi D., Tsukahara T., Matsumoto M., Amagase K., Iwata K., Nakamura A. (2017). NOX1/NADPH oxidase expressed in colonic macrophages contributes to the pathogenesis of colonic inflammation in trinitrobenzene sulfonic acid-induced murine colitis. J. Pharmacol. Exp. Ther..

[40] Welak S.R., Rentea R.M., Teng R.J., Heinzerling N., Biesterveld B., Liedel J.L., Pritchard K.A., Fredrich K.M., Gourlay D.M. (2014). Intestinal NADPH oxidase 2 activity increases in a neonatal rat model of necrotizing enterocolitis. PLoS ONE.

[41] Smith P.D., Smythies L.E., Mosteller-Barnum M., Sibley D.A., Russell M.W., Merger M., Sellers M.T., Orenstein J.M., Shimada T., Graham M.F. (2001). Intestinal macrophages lack CD14 and CD89 and consequently are down-regulated for LPS- and IGA-mediated activities. J. Immunol..

[42] Smythies L.E., Sellers M., Clements R.H., Mosteller-Barnum M., Meng G., Benjamin W.H., Orenstein J.M., Smith P.D. (2005). Human intestinal macrophages display profound inflammatory anergy despite avid phagocytic and bacteriocidal activity. J. Clin. Investig..

[43] Smith P.D., Smythies L.E., Shen R., Greenwell-Wild T., Gliozzi M., Wahl S.M. (2011). Intestinal macrophages and response to microbial encroachment. Mucosal Immunol..

[44] Rugtveit J., Haraldsen G., Hogasen A.K., Bakka A., Brandtzaeg P., Scott H. (1995). Respiratory burst of intestinal macrophages in inflammatory bowel disease is mainly caused by CD14^+^L1^+^ monocyte derived cells. Gut.

[45] Roberts P.J., Riley G.P., Morgan K., Miller R., Hunter J.O., Middleton S.J. (2001). The physiological expression of inducible nitric oxide synthase (iNOS) in the human colon. J. Clin. Pathol..

[46] Denning T.L., Wang Y.C., Patel S.R., Williams I.R., Pulendran B. (2007). Lamina propria macrophages and dendritic cells differentially induce regulatory and interleukin 17-producing t cell responses. Nat. Immunol..

[47] Hadis U., Wahl B., Schulz O., Hardtke-Wolenski M., Schippers A., Wagner N., Muller W., Sparwasser T., Forster R., Pabst O. (2011). Intestinal tolerance requires gut homing and expansion of foxp3+ regulatory t cells in the lamina propria. Immunity.

[48] Zigmond E., Bernshtein B., Friedlander G., Walker C.R., Yona S., Kim K.-W., Brenner O., Krauthgamer R., Varol C., Müller W. (2014). Macrophage-restricted interleukin-10 receptor deficiency, but not IL-10 deficiency, causes severe spontaneous colitis. Immunity.

[49] Cheroutre H. (2004). Starting at the beginning: New perspectives on the biology of mucosal T cells. Annu. Rev. Immunol..

[50] Kang S.G., Lim H.W., Andrisani O.M., Broxmeyer H.E., Kim C.H. (2007). Vitamin a metabolites induce gut-homing foxp3+ regulatory T cells. J. Immunol..

[51] Rani R., Smulian A.G., Greaves D.R., Hogan S.P., Herbert D.R. (2011). TGF-beta limits IL-33 production and promotes the resolution of colitis through regulation of macrophage function. Eur. J. Immunol..

[52] Hershberg R.M., Framson P.E., Cho D.H., Lee L.Y., Kovats S., Beitz J., Blum J.S., Nepom G.T. (1997). Intestinal epithelial cells use two distinct pathways for hla class ii antigen processing. J. Clin. Investig..

[53] Bland P. (1988). MHC class II expression by the gut epithelium. Immunol. Today.

[54] Hershberg R.M., Mayer L.F. (2000). Antigen processing and presentation by intestinal epithelial cells—Polarity and complexity. Immunol. Today.

[55] Pascual-Reguant A., Bayat Sarmadi J., Baumann C., Noster R., Cirera-Salinas D., Curato C., Pelczar P., Huber S., Zielinski C.E., Lohning M. (2017). TH17 cells express ST2 and are controlled by the alarmin IL-33 in the small intestine. Mucosal Immunol..

[56] Bayerl S.H., Niesner R., Cseresnyes Z., Radbruch H., Pohlan J., Brandenburg S., Czabanka M.A., Vajkoczy P. (2016). Time lapse in vivo microscopy reveals distinct dynamics of microglia-tumor environment interactions-a new role for the tumor perivascular space as highway for trafficking microglia. Glia.

[57] Rinnenthal J.L., Börnchen C., Radbruch H., Andresen V., Mossakowski A., Siffrin V., Seelemann T., Spiecker H., Moll I., Herz J. (2013). Parallelized TCSPC for dynamic intravital fluorescence lifetime imaging: Quantifying neuronal dysfunction in neuroinflammation. PLoS ONE.

[58] Farache J., Koren I., Milo I., Gurevich I., Kim K.-W., Zigmond E., Furtado G.C., Lira S.A., Shakhar G. (2013). Luminal bacteria recruit cd103(+) dendritic cells into the intestinal epithelium to sample bacterial antigens for presentation. Immunity.

[59] Cossarizza A., Chang H.D., Radbruch A., Akdis M., Andra I., Annunziato F., Bacher P., Barnaba V., Battistini L., Bauer W.M. (2017). Guidelines for the use of flow cytometry and cell sorting in immunological studies. Eur. J. Immunol..

